# Analysis of Epicardial Adipose Tissue Texture in Relation to Coronary Artery Calcification in PCCT: The EAT Signature!

**DOI:** 10.3390/diagnostics14030277

**Published:** 2024-01-27

**Authors:** Peter Mundt, Alexander Hertel, Hishan Tharmaseelan, Dominik Nörenberg, Theano Papavassiliu, Stefan O. Schoenberg, Matthias F. Froelich, Isabelle Ayx

**Affiliations:** 1 Department of Radiology and Nuclear Medicine, University Medical Center Mannheim, Heidelberg University, 68167 Mannheim, Germany; peter.mundt@uni-ulm.de (P.M.); alexander.hertel@medma.uni-heidelberg.de (A.H.); hishan.tharmaseelan@medma.uni-heidelberg.de (H.T.); dominik.noerenberg@medma.uni-heidelberg.de (D.N.); stefan.schoenberg@umm.de (S.O.S.); matthias.froelich@medma.uni-heidelberg.de (M.F.F.); 2 First Department of Internal Medicine-Cardiology, University Medical Centre Mannheim, and DZHK (German Centre for Cardiovascular Research), Partner Site Heidelberg/Mannheim, 68167 Mannheim, Germany

**Keywords:** radiomics, texture analysis, photon-counting computed tomography, epicardial adipose tissue, coronary artery calcium score

## Abstract

(1) Background: Epicardial adipose tissue influences cardiac biology in physiological and pathological terms. As it is suspected to be linked to coronary artery calcification, identifying improved methods of diagnostics for these patients is important. The use of radiomics and the new Photon-Counting computed tomography (PCCT) may offer a feasible step toward improved diagnostics in these patients. (2) Methods: In this retrospective single-centre study epicardial adipose tissue was segmented manually on axial unenhanced images. Patients were divided into three groups, depending on the severity of coronary artery calcification. Features were extracted using pyradiomics. Mean and standard deviation were calculated with the Pearson correlation coefficient for feature correlation. Random Forest classification was applied for feature selection and ANOVA was performed for group comparison. (3) Results: A total of 53 patients (32 male, 21 female, mean age 57, range from 21 to 80 years) were enrolled in this study and scanned on the novel PCCT. “Original_glrlm_LongRunEmphasis”, “original_glrlm_RunVariance”, “original_glszm_HighGrayLevelZoneEmphasis”, and “original_glszm_SizeZoneNonUniformity” were found to show significant differences between patients with coronary artery calcification (Agatston score 1–99/≥100) and those without. (4) Conclusions: Four texture features of epicardial adipose tissue are associated with coronary artery calcification and may reflect inflammatory reactions of epicardial adipose tissue, offering a potential imaging biomarker for atherosclerosis detection.

## 1. Introduction

Epicardial adipose tissue (EAT) surrounds the heart and—besides its protective properties—influences cardiac physiology [1]. While playing a role in regular physiologic processes of the heart, it has also been found to be linked to pathologies such as atrial fibrillation and coronary artery calcification (CAC) [2,3], of which local inflammatory and toxic effects could influence the latter, as these have been described for other adipose tissues [4]. EAT may also offer further diagnostic possibilities, as it has been found to significantly decrease mass in patients with non-ischemic dilated cardiomyopathy and its mass to significantly correlate with left-ventricular mass and volume in these patients [5].

According to the World Health Organization, ischemic heart disease remains the leading cause of death worldwide [6], making the identification and treatment of patients at risk a highly important task of modern medicine. These insights and the technological improvements in computed tomography (CT) led to the readjustment of guidelines by the European Society of Cardiology (ESC) and strengthened the role of cardiac CT angiography as the preferable diagnostic tool for patients, who present with a low to intermediate pretest probability for coronary artery disease (CAD) [7]. In line with these guidelines, the recently published DISCHARGE study could demonstrate that patients presenting with stable chest pain and intermediate pretest probability of CAD suffered more frequently from major procedure-related complications when receiving an initial invasive coronary angiography instead of an initial CT, whereas the risk for a major adverse cardiovascular event (MACE) stayed the same [8]. Apart from detecting coronary artery stenosis, cardiac CTs offer the possibility to measure the CAC [9]. As CAC is a major risk factor for cardiovascular diseases (CVD), measuring it in the form of the coronary artery calcium score (CACS) has proven to be a reliable way for risk stratification of such respective patients [10]. In clinical practice, the Agatston score is widely used to estimate the extent of calcification in the main coronary arteries [11] based on the extent and density of CAC. It can not only be used to estimate the risk of MACE in the future but has also been linked to various other diseases such as dementia and even cancer [12].

Additionally, a risk profile can be created using the Agatston score to calculate a so-called cardiac age and offers the opportunity to define the probability of the development of an obstructive CAD event within the next 10 years [13]. A cut-off value of 100 was chosen as a reference point for the occurrence of most adverse cardiovascular events in CAD [13]. To find ways to extend the diagnostics of such patients at risk, research on EAT properties with potential diagnostic use has increased over the last few years. While these studies have often been based on the volume [14,15] of the adipose tissue, more recent studies have conducted radiomics examinations of perivascular intrathoracic adipose tissue with first promising results [16,17].

Radiomics is a field of medical image analysis that has been rapidly evolving. It analyses pixel-based data within the image beyond the limitations of the examiner’s physiological capabilities. The focus lies on the analysis of a region/volume of interest’s (VOI/ROI) quantitative metrics, so-called features, such as texture and shape, and may offer a feasible extension of medical imaging [18]. Supported by a sufficient database, these features are minable, meaning it might be possible to identify new features as potential characteristic markers of certain pathologies. Therefore, radiomics features may have the potential to serve as future imaging biomarkers [19]. Having been mainly established in oncology research [20,21], radiomics analysis is an increasingly upcoming method in cardiac imaging research [22] with promising results [16,23,24]. Nevertheless, radiomics analysis is known for its lack of reproducibility and comparability, which hampers its use in clinical routine [25]. For a stable and comparable radiomics analysis, excellent image quality and spatial resolution have been defined as the underlying foundation [26].

A promising tool to address this obstacle might be the recently introduced Photon-Counting computed tomography (PCCT). This revolutionary technology converts in comparison to traditional energy-integrating detector CTs (EICT) the incoming photon directly into an electric signal without the intermediate step of converting it into visible light. Through these changes in detector technologies PCCT scanners offer an increased spatial and temporal resolution, as well as a higher contrast-to-noise ratio and lower beam-hardening artifacts [27,28]. These advancements in the PCCT scanners may provide a promising improvement to the field of radiomics. A recent study has proven the high stability of radiomics features in organic phantoms and might pave the way for the final use of cardiac radiomics analysis in clinical routine [28].

Hence, this study aims to investigate a possible correlation between EAT texture features and the extent of CAC using PCCT images and to possibly identify potential biomarkers for the detection of inflammatory reactions in EAT leading to CAC.

## 2. Materials and Methods

### 2.1. Study Design

This retrospective single-centre study enrolled patients with suspected or known CAC who had the clinical indication for electrocardiography (ECG) gated cardiac CT between December 2021 and March 2022 following the current guidelines [7]. The enrolled patients were screened for possible inhomogeneities affecting the EAT by a board-certified radiologist with 10 years of experience in cardiothoracic imaging (*n* = 0). Exclusions were made on the grounds of previous stent implantation (*n* = 5), and grave image artefacts due to motion (*n* = 3). The study had the approval of the institutional review board and local ethics committee (ID 2021-659) and was conducted in accordance with the Declaration of Helsinki.

### 2.2. Chest CT Imaging Protocol

All included patients were scanned on a first-generation whole-body dual-source PCCT system (NAEOTOM Alpha; Siemens Healthcare GmbH, Forchheim, Germany) using a prospective ECG-gated sequential mode with a tube voltage of 120 kV and automatic dose modulation with a CARE keV BQ setting of 64. The effective gantry rotation time was 0.25 s.

Depending on the heart rate and the absence of contraindications, patients received 5–10 mg of intravenous metoprolol to ensure a sufficiently low heart rate as well as 0.4–0.8 mg of sublingual nitroglycerin. All patients underwent a non-contrast-enhanced cardiac CT for evaluation of CAC followed by a contrast-enhanced cardiac CT of the coronary arteries, which was not part of this study.

### 2.3. Chest CT Imaging Analysis

**Figure 1:**
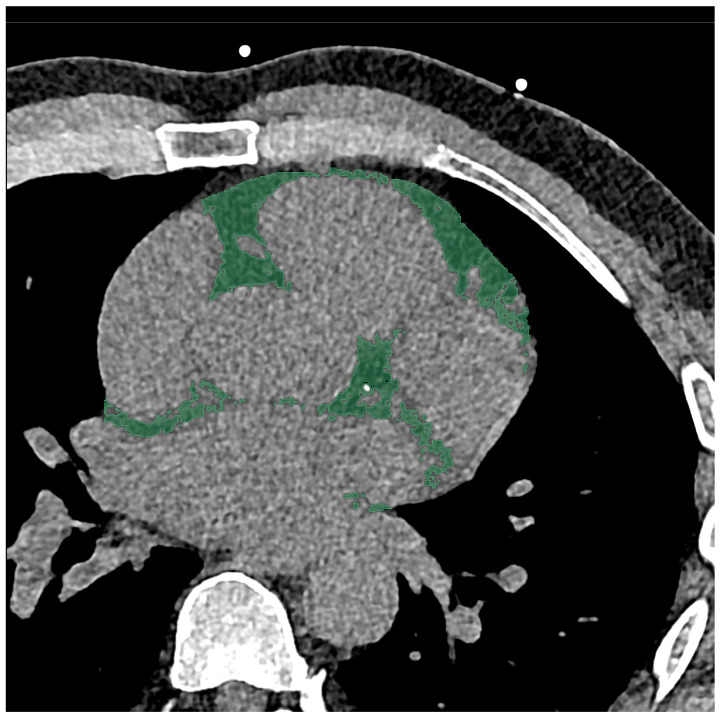
Segmentation of the epicardial adipose tissue was performed on an axial view CT scan with a slice thickness of 2 mm. On this scan of a 55-year-old male, the area of segmentation is marked in green.

A soft vascular kernel (Bv40) was used to reconstruct axial non-contrast-enhanced images with a slice thickness of 2 mm (2 mm increment). The data was then anonymized, exported, and stored using digital imaging and communications in medicine (DICOM) format. For segmentation with a dedicated segmentation tool (3D Slicer, Version 4.11) [29], the aforementioned files were converted to the Neuroimaging Informatics Technology Initiative (NIFTI) format. EAT was segmented manually by a medical student and reviewed by a board-certified radiologist with 10 years of clinical experience in cardiovascular imaging and 6 years of experience in segmentation. The entire EAT was included in the segmentation, using a CT-attenuation threshold of −190 to −30 Hounsfield units (HU), in line with the literature [30]. The middle of the pulmonary trunk was used as a cranial border for the segmentation, with the caudal border being the contact of the apex and diaphragm. An example of segmentation is shown in Figure 1.

Calculation of the Agatston score was performed using dedicated software (syngo. via, Version VA50 SW, Siemens Healthcare GmbH, Forchheim, Germany) based on axial non-contrast-enhanced CT scans with 3 mm slice thickness and a quantitative Qr36 kernel. The population was then split into three groups depending on the Agatston score and hence the severity of CAC; Agatston score 0 (Agatston 0), Agatston score 1–99 (Agatston 1), and Agatston Scores of 100 or above (Agatston 2).

### 2.4. Radiomics Feature Extraction and Statistical Analysis

Radiomics features of the EAT were extracted further by using an imaging biomarker standardisation initiative definition (IBSI)-based Python package (pyradiomics, Version 3.0.1) [31]. The first and second order features grey level co-occurrence matrix (glcm), grey level dependence matrix (gldm), grey level size zone matrix (glszm), grey level run length matrix (glrlm), and neighbouring grey tone difference matrix (ngtdm) were extracted for each VOI. Additionally, EAT density and volume were also calculated.

Statistical analyses were performed using R (R Statistics, Version 4.1.2, R Core Team, Vienna, Austria) [32], and RStudio (Version 2021.09.2, Boston, MA, USA) [33].

Mean and standard deviation (SD) were calculated for all quantitative parameters, and categorical variables were summarised in percentages. All radiomics features were normalised using z-score:

z = ((X − μ))/σ,

µ being the mean and σ the feature standard deviation.

For the correlation of feature calculation, Pearson correlation coefficients were used. Using the ComplexHeatmap package in R, features were visualised in boxplots and heatmaps. Within each Agatston score group hierarchical clustering was performed. For feature selection by the calculation of feature importance, a permutation-based Random Forest (RF) classifier was used with the R Boruta package. Anova analysis was then performed to compare groups using SPSS (IBM Corp. Released 2021. IBM SPSS Statistics for Windows, Version 28.0. Armonk, NY, USA: IBM Corp.) [34].

## 3. Results

### 3.1. Patient Collective

**Figure 2:**
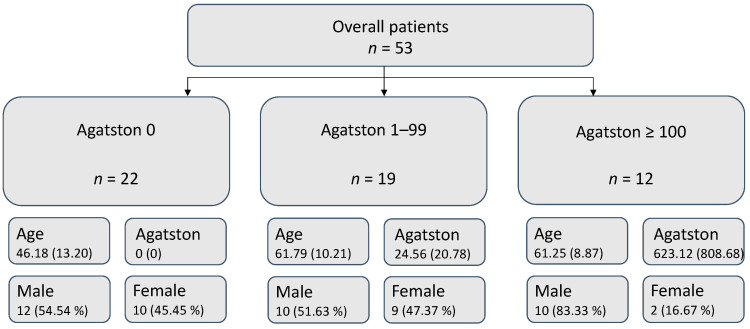
Patient collective overview. Mean and (SD) are given for continuous variables.

In total, 53 suitable patients (32 male, 21 female, mean age 57, range 21 to 80 years) were enrolled in this study. Due to the out-patient setting, in which most cases were acquired and the retrospective nature of this study, further clinical data could not be sufficiently obtained (Table 1). The patient’s characteristics depending on the severity of CAC are summarised in Figure 2.

### 3.2. Cluster Analysis

**Figure 3:**
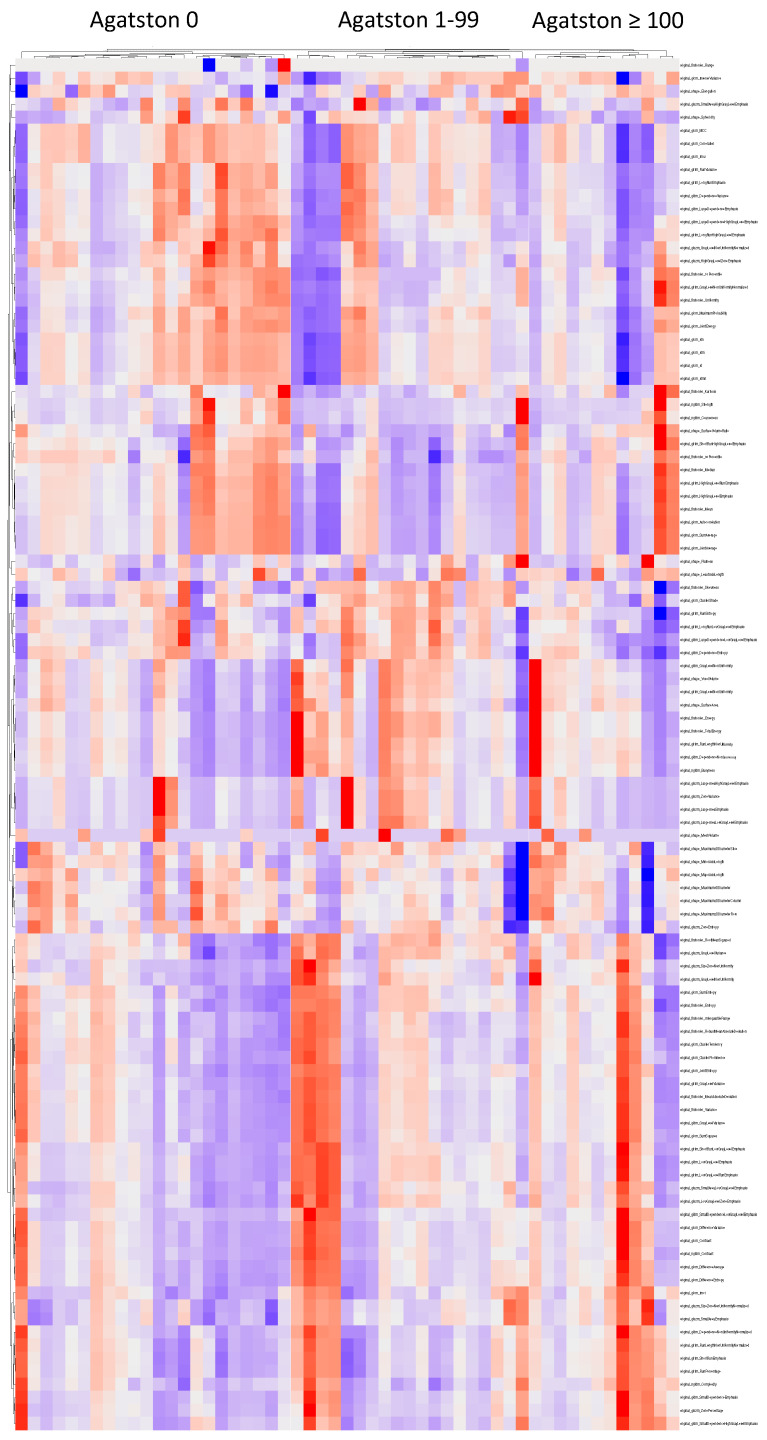
Clustered Heatmap of radiomics features of EAT.

After standardisation, hierarchical clustering of the extracted EAT features of each patient was performed in addition to clustering within each of the Agatston score groups (Figure 3).

### 3.3. Feature Selection

**Figure 4:**
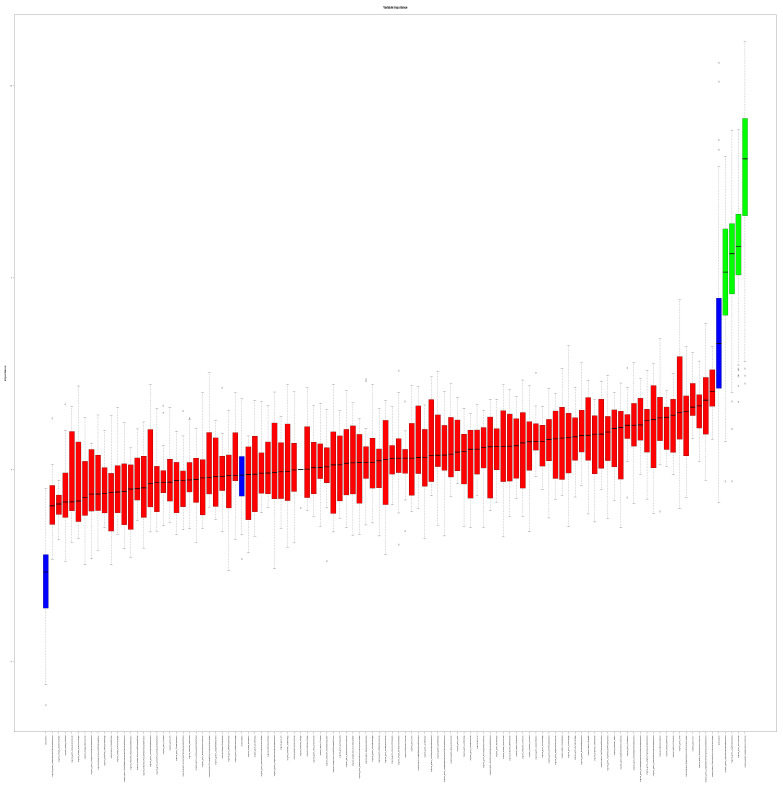
Random Forest feature selection (differentiating features in green).

Based on the EAT texture, important features were selected by the RF Boruta feature selection on the Agatston score groups Agatston 0 and Agatston ≥ 100. Four different features, namely “original_glrlm_LongRunEmphasis”, “original_glrlm_RunVariance”, “original_glszm_HighGrayLevelZoneEmphasis”, and “original_glszm_SizeZoneNonUniformity” were identified to be associated with differences in Agatston scores (Figure 4).

### 3.4. Internal Validation

**Figure 5:**
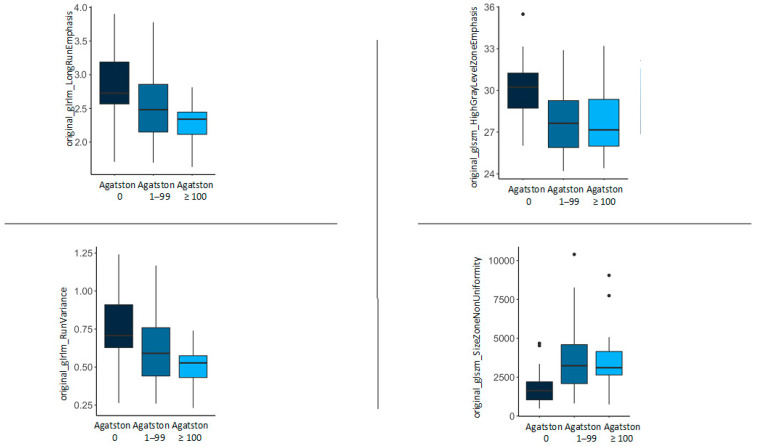
Feature distribution within the Agatston score groups of “original_glrlm_LongRunEmphasis”, “original_glszm_HighGrayLevelZoneEmphasis”, “original_glrlm_RunVariance”, and “original_glszm_SizeZoneNonUniformity”.

The four selected features were investigated in the group of Agatston score 1–99 for internal validation purposes. Except “original_glszm_HighGrayLevelZoneEmphasis” (Agatston score 0/1–99/≥100 30.16/27.68/27.83, *p* = 0.003), the group Agatston score 1–99 settled in between the other two groups, supporting the expected changes of EAT with increasing Agatston score with corresponding values for “original_glrlm_LongRunEmphasis” (2.82/2.55/2.27 *p* = 0.013), “original_glrlm_RunVariance” (0.74/0.62/0.50 *p* = 0.013) and “original_glszm_SizeZoneNonUniformity” (1893.05/3815.24/3817.49 *p* = 0.005). These results are listed in Table 2 and visualised in Figure 5.

### 3.5. Additional Imaging Features

In addition to the aforementioned features, EAT volume and density were also investigated in the form of “original_firstorder_Mean” (density) and “original_shape_MeshVolume” (volume). The density measurement included the whole epicardial adipose tissue as extracted from the segmentation (Figure 1). The corresponding results are listed in Table 2.

### 3.6. Feature Description

Two of the identified features are “gray level size zone matrix” features. A Gray Level Size Zone Matrix (glszm) quantifies gray level zones in an image. A gray level zone is the number of connected voxels that share the same gray level intensity [35]. The first “glszm” feature, “original_glszm_HighGrayLevelZoneEmphasis” is a measurement for the distribution of lower gray-level zones. Higher values are indicative of a greater proportion of lower gray-level values and size zones [35]. The second “glszm” feature, “original_glszm_SizeZoneNonUniformity” measures the variability of size zone volumes. Lower values indicate more homogeneity within the size zone volumes [35]. The other two identified features are “gray level run length matrix” features. A Gray Level Run Length Matrix (glrlm) quantifies gray level runs, which are the length of consecutive pixels with the same gray level value [36]. The feature “original_glrlm_LongRunEmphasis” is a measurement for the distribution of long run lengths. Greater values indicate longer runs and a more coarse texture [36]. Lastly, “original_glrlm_RunVariance” measures the variance in gray level intensity in the runs [36].

## 4. Discussion

By identifying four radiomics EAT texture features that are associated with differences in Agatston score, this study implies a possible connection between CAC and the texture of EAT. Additionally, three out of these four features were dependent on the amount of CAC, strengthening the thesis of a connection between CAC and EAT texture. The presentation of “original_glrlm_LongRunEmphasis” with higher values in the group of Agatston score 0 and lower values in the group of Agatston score ≥ 100 together with “original_glszm_SizeZoneNonUniformity” with lower values in the Agatston score 0 group and higher values in the Agatston score ≥ 100 group indicates a more heterogeneous but finer EAT structure with rising Agatston score and more homogeneous but coarse EAT structure in patients without CAC. Additionally, “original_glszm_HighGrayLevelZoneEmphasis” has shown higher values in the group without CAC than in the two groups with CAC, implying a lower proportion of higher gray-level values in EAT in the groups with an Agatston score > 0. The mean density measured in the three groups was also highest in the group with no CAC (mean −79.11 HU), which further supports the theory that decreasing EAT density is associated with increased CACS. A linear correlation between EAT density and CAC could not be shown however, as there was no significant difference between the patients with CAC (Agatston score 1–99: mean −86.38 HU; Agatston score ≥ 100: mean −81.73 HU). The values in this preliminary study could be explained by adipocyte hypertrophy, which is linked to inflammation of the adipose tissue [2], leading to a lower proportion of high-density values and more heterogeneous, less coarse structure through possibly underlying additional inflammatory changes in patients with CAC.

The correlation between EAT density and cardiovascular risk factors as well as adverse cardiovascular events is still the subject of scientific debate. In line with our study, there are findings on lower EAT density being associated with an increase in risk factors and CACS, as presented by Franssens et al. [9], They investigated the correlation between CAC and EAT attenuation in 2017 in 140 patients (100 male, 40 female) from the ongoing SMART study, grouped by Agatston score (0/1–100/101–400/>400) and found lower EAT attenuation to be associated with higher CAC. In their study, a decrease in EAT attenuation by one SD was associated with increased odds of 1.77 (95% CI 1.18–2.66) for a higher CAC density after adjustment for age, sex, and coronary artery bypass graft history. They concluded that independent of BMI or EAT volume, lower EAT attenuation is associated with higher amounts of CAC in men at high risk of or with cardiovascular disease [9].

Pandey et al. [37] found EAT attenuation to be significantly lower in patients with obstructive CAD when they investigated the association between EAT volume, EAT attenuation, obstructive CAD, and high-risk plaque features in 2020. They enrolled 255 patients (132 male, 123 female) with atypical chest pain who underwent coronary CT angiography, using non-contrast CT images to semi-automatically assess EAT volume and attenuation. A significant association between EAT density and EAT volume could not be found (*p* = 0.0576) and EAT volume did not differ significantly between patients with obstructive CAD and those without (median 116.98cc, IQR 84.08–139.365 vs. median 102.65cc, IQR 79.33–131.02, *p* = 0.2960). EAT attenuation, however, was found to be significantly lower in patients with obstructive CAD than in those without (median −86 HU, IQR −88 to −82 vs. median −84 HU, IQR −87 to −82, *p* = 0.0486). Furthermore, it showed a significant association with obstructive CAD in univariate analysis (unadjusted OR = 0.9 (0.81–0.99), 95% CI, *p* = 0.0248). They concluded EAT attenuation to be an independent predictor of obstructive CAD as well as of high-risk plaque features, as it was significant on a multivariate logistics regression analysis for predictors of obstructive CAD (adjusted OR = 0.89 (0.8–0.99), 95% CI, *p* = 0.033) and high-risk plaque features (adjusted OR = 0.72 (0.61–0.85), 95% CI, *p* = 0.0001), and recommended it to be used in the estimation of the pretest probability of both [37].

However, there have also been findings on increased EAT density associated with increased risk of CAD as, for example, pointed out by Liu et al. [38], in 2019. A total of 614 patients (61% male) who underwent CT angiography were enrolled in their study. EAT volume and attenuation were manually traced and calculated from contrast-enhanced CT angiography images. The mean EAT attenuation was −73.53 ± 5 HU and the mean Agatston score was 127 ± 330. After multivariable adjustment, they found EAT attenuation to be associated with the Agatston score with an odds ratio of 1.21 (1.05 to 1.4, 95% CI, *p* = 0.01) and concluded higher EAT attenuation to be associated with a higher risk of CAD [38].

Gao et al. [39], came to a similar conclusion in 2022 when they investigated the association between EAT density and coronary plaque characteristics. They examined a total of 240 patients (55.83% male) with chest pain or precardiac symptoms who underwent coronary CT angiography. Based on the results of this coronary CT angiography, the cohort was split into two groups, one group with high-risk plaques (133 patients, 83 males) and one without high-risk plaques (107 patients, 50 males). Segmentation was carried out semi-automatically on the original coronary CT angiography dataset and the EAT volume and average density were automatically calculated. The mean EAT density was −101.24 ± 15.90 for the high-risk plaque group and −107.28 ± 14.79 for the non-high-risk plaque group. After adjustment for age, sex, family history of coronary heart disease, and diabetes, they found both EAT volume and EAT density to be independent predictors of high-risk plaques. The risk increase of high-risk plaques was 3.120 (2.147–4.533, CI 95% *p* = 0.000) times the risk of non-high-risk plaques per 1 SD increase in EAT density and 1.499 (1.068–1.965, CI 95% *p* = 0.017) times per 1 SD increase in EAT volume. When comparing the predictive value of EAT density and volume for high-risk plaques, they found EAT density to predict the presence of high-risk plaques better than EAT volume when performing a ROC analysis (AUC = 0.761 vs. 0.606, CI 0.701–0.822 vs. 0.534–0.678, *p* = 0.000/0.005) [39].

Increased EAT volume has been linked to coronary and myocardial diseases [2] and CAC [3,15] in several studies. Cosson et al. [3] investigated whether the volume of EAT was independently associated with CAC in a study population of 409 patients (218 male, 191 female) with diabetes in 2021. CAC and EAT were calculated using non-contrast CT images and patient data such as tobacco consumption, smoking history, ethnicity, patient history, and biomarkers were collected. They found EAT volume, to be positively associated with, amongst other parameters, an Agatston score of ≥100 and found patients with an Agatston score of ≥100 to be older, more likely to have type 2 diabetes, more likely to have diabetes-related complications, and a more extensive smoking history. After adjustment for all of these parameters, as well as gender and BMI, they found that EAT volume was independently associated with an Agatston score ≥ 100 in multivariable analysis [3].

Coming to a similar conclusion in 2022, Liu et al. [15], investigated the relationship between EAT volume and CAC as well as pericardial adipose tissue volume and CAC in 523 patients (289 male, 234 female) who underwent coronary CT angiography. They divided both subgroups depending on the extent of CAD, by defining the CAD group as coronary artery stenosis ≥ 50% and the non-CAD group as a stenosis of <50% or no stenosis (respective mean EAT volumes 132.0 cm^3^/102.0 cm^3^, *p* < 0.001) and found EAT volume to be higher in patients with an Agatston score ≥ 100 [15]. These results are in line with our study, as we also found EAT volume to be lower in the Agatston score 0 group (mean 257,339.30 mm^3^) than in patients with CAC (Agatston score 1–99: 386,997.04 mm^3^/Agatston score ≥ 100: 333,642.30 mm^3^), implying an adverse association of EAT volume and CAC, although no linear correlation between the CAC severity and EAT volume could be found.

As a well-established tool in oncology research, the use of radiomics in the field of cardiovascular imaging, especially regarding cardiac adipose tissues and their texture, may offer extended diagnostic value in the future. In 2023, Kahmann et al. [40] investigated changes in pericoronary adipose tissue in patients with hypercholesterolemia using radiomics analysis. The pericoronary adipose tissue of the left and right coronary arteries were segmented manually. Radiomics features were extracted with pyradiomcis on a test collective of 48 patients (20 male, 28 female) and validated with a validation collective of 18 patients (12 male, 6 female) with each collective being divided into two groups by the presence of hypercholesterolemia or the lack thereof. Using Random Forest feature selection, the most important features for differentiation between patients with and without hypercholesterolemia were identified and the four most important features were investigated in the validation collective. They identified two radiomics features to differ between patients with hypercholesterolemia and those without, offering a potential imaging biomarker [40].

While examining different cardiac adipose tissues regarding different variables, their findings in line with our findings outline the feasibility and applicability of radiomics texture analyses in cardiovascular imaging. At the same time, their findings indicate that different risk factors seem to affect cardiac adipose tissues, which needs to be addressed in future studies.

The use of radiomics in the examination of perivascular adipose tissue in connection to coronary calcification has been shown to be feasible and of potential use, as shown in a study of our institution from 2023. In that study, the possible correlation between the extent of CAC and periaortic adipose tissue texture was investigated using radiomics. The thoracic periaortic adipose tissue of 55 patients (34 male, 21 female) who had undergone cardiac CT was segmented manually on non-contrast-enhanced CT images, and radiomics features were extracted using pyradiomics. The patient population was split into three groups by their respective extent of CAC (Agatston score 0/1–99/≥100). Radiomics features for differentiation based on the periaortic adipose tissue textures were identified using Random Forest-based feature selection on the Groups of Agatston score 0 and ≥100 and investigated in the Group of 1–99 for internal validation. It was found that two radiomics features differed between the groups, one of them significantly [17]. While the investigated adipose tissues as well as the identified radiomics features differ from our study, it nevertheless supports our findings, as it underlines the feasibility of distinguishing between patients with and without CAC using radiomics texture features of adipose tissues, indicating a potential underlying diffuse fibrotic or inflammatory reaction, which can be detected by texture analysis.

In 2021 Zheng et al. [16], conducted a study investigating the radiomics texture analysis of peri-coronary adipose tissue in the diagnosis of CAD on a cohort of 67 (38 male, 29 female) patients with CAD, splitting the patients into two groups by the presence of a significant lesion in the right coronary artery or the lack of such. Out of 1218 radiomics features, wavelet-based texture features differed significantly between both groups (highest Area Under Curve 0.78), hence they found the texture analysis to help predict plaque existence [16]. Although this study investigated EAT rather than peri-coronary adipose tissue, this is supported by our data, providing texture features that allow differentiation between patients with and without CAC. The differences in the study setting ranging from different CT scanners (energy-integrating detector CT versus PCCT) to different feature extraction methods (wavelet filtering vs. original) outline the problem of radiomics analysis in clinical routine through the lack of reproducibility. Ayx et al., have pointed out that there are noticeable differences regarding radiomics features, especially in higher-order features, between EICT scanners and PCCT scanners [41]. However, there is evidence that suggests that PCCT shows high feature stability [28] and might therefore be superior for radiomics feature analysis and lead to implementation in clinical routine.

The results of this study must be interpreted within the scope of the following limitations: This study is a single-centre retrospective study. Although the newly established PCCT offers increased image quality [42] and is more stable in texture analysis [28], the number of patients was severely limited by the selection criteria and the limited number of patients that were scanned on a PCCT. Different clinical risk factors such as e.g., hypercholesterolemia as mentioned above [40] seem to influence the texture of cardiac adipose tissues, which must be considered in future radiomics studies of adipose tissue texture. Due to the outpatient setting and the retrospective nature of this study, we were unable to account for sufficient clinical parameters. Furthermore, the sex distribution in the group of Agatston score ≥ 100 is unbalanced, with 83% male patients. Additionally, the age distribution differs between the group with an Agatston score of 0 and the groups with an Agatston score of 1–99/≥100. Further investigations with a more even sex and age distribution, as well as sufficient clinical data, should be performed, ideally in a prospective multi-centre setting with a larger patient population. To better understand the underlying histopathological mechanism additional studies must include a histological examination of EAT in patients with various degrees of CAC.

## 5. Conclusions

In conclusion, the results of this study imply a connection between the texture of EAT and CAC. We found a pattern of differences in EAT texture between patients afflicted by CAC and those without it in the form of four radiomics texture features. It appears that patients affected by CAC present with a more heterogeneous but less coarse EAT texture than those free of CAC. Further research is needed to validate these findings, but the preliminary findings of this study suggest that the use of radiomics features poses a viable aid in the detection of changes in EAT texture and the presumably subsequent development of CAC. Supported by future studies with sufficient datasets, radiomics texture features of EAT may present possible biomarkers for CAC and aid in its assessment and identification in the future.

## Figures and Tables

**Table 1:** Available clinical data for the respective groups.

	Overall Patients	Agatston 0	Agatston 1–99	Agatston ≥ 100
*n*	53	22	19	12
Patients with known cardiovascular risk factors	24	7	10	7
Diabetes	1	1	0	0
Hypertension	19	5	9	5
Dislipidemia	4	3	1	0
Nicotine abuse	5	1	2	2

**Table 2:** Mean (standard deviation) given for relevant features.

Feature	Agatston 0	Agatston 1–99	Agatston ≥ 100	*p*-Value
original_glrlm_LongRunEmphasis	2.82 (0.51)	2.55 (0.56)	2.27 (0.34)	0.013
original_glrlm_RunVariance	0.74 (0.23)	0.62 (0.25)	0.50 (0.15)	0.013
original_glszm_HighGrayLevelZoneEmphasis	30.16 (2.32)	27.68 (2.42)	27.83 (2.43)	0.003
original_glszm_SizeZoneNonUniformity	1893.05 (1156.82)	3815.24 (2487.78)	3817.49 (2390.38)	0.005
Density (HU)	−79.11 (5.81)	−86.38 (6.14)	−81.73 (8.06)	0.003
Volume (mm^3^)	257,339.30 (109,963.91)	386,997.04 (162,348.54)	333,642.30 (203,406.17)	0.033

## Data Availability

The data presented in this study are available on request from the corresponding author.
